# Supplementary material for: Are carbon emissions trading and green financial instruments synergistic? -Comprehensive quantitative research based on content analysis
Source: PLoS One. 2024 Mar 7;19(3):e0298601. doi: 10.1371/journal.pone.0298601 (PMC10919601; doi:10.1371/journal.pone.0298601)
Supplement: S1 Data — (ZIP) [file pone.0298601.s001.zip › Data/地级市Z_F工作报告.zip!/─■╧─/╬Γ╓╥╩╨/└┤╫╘╣1⁄2╓┌║┼í╛┬φ┐╦╩2╛▌═°í┐_2013.docx]

各位代表：

　　现在，我代表市人民政府，向大会作政府工作报告，请予审议，并请市政协委员和其他列席人员提出意见。

五年工作回顾

　　本届政府的五年，吴忠发展正处在一个较为困难和关键的时期：既要在国际金融危机的复杂形势下谋划科学发展，又要在区划调整后的市情中实现跨越发展。面对这样一个局面，按照“一年一个新变化、三年上个新台阶、五年有个大发展”的目标和要求，在自治区党委、政府和市委的坚强领导下，在市人大、政协的监督支持下，本届政府顽强拼搏，埋头苦干，推进大开放、实施大开发、加快大建设，实现了大跨越，兑现了对全市人民的郑重承诺！这五年，是吴忠经济发展最快、城乡变化最大、群众得到实惠最多、民族更为团结、社会更加和谐的五年，也是各族人民充满自信、引以自豪的五年！

　　——五年来，狠抓第一要务，综合实力迈上新台阶。主要经济指标实现翻番。预计2012年完成地区生产总值307亿元，是2007年的2.1倍，年均增长13%；地方公共财政预算收入27亿元，是2007年的2.1倍，年均增长17%；全社会固定资产投资380亿元，比上年增长46%，累计完成1176亿元，是上个五年的4倍；城镇居民人均可支配收入、农民人均纯收入分别由2007年的9680元、3611元增加到17800元和6300元，年均分别增长13%和12%。截止11月底，地区生产总值、财政预算收入、固定资产投资增幅均居全区五市第一。县域经济竞相发展。利通区经济社会多项指标位于全市前列；红寺堡区综合实力逐年提升进位；青铜峡市跃升西部百强县第29位；盐池县综合实力位居全区山区县之首；同心县主要经济指标全部实现翻番。

　　——五年来，紧扣主题主线，三次产业实现新突破。加快推进新型工业、现代农业和第三产业发展，不断优化经济结构，切实转变发展方式，产业支撑带动能力明显增强。

　　工业经济逆势突破。预计2012年实现规模以上工业增加值98.5亿元，按可比价比2007年增长1.1倍，年均增长16%。“两城五园”成效显著。五年共实施重点项目330个，完成投资427亿元，分别占全市工业比重的87%和83%。能源化工、装备制造、食品加工、乳制品、烟草等产业不断壮大，风光电装机容量、煤炭开采能力分别达到137万千瓦和680万吨，是2007年的6.8倍和7.5倍。培育年销售收入过亿元、10亿元企业分别达到61家和8家，是2007年的3倍和2倍。自主创新步伐加快。建成国家级、自治区级企业技术研发中心6家、市级19家，培育“中国驰名商标”8个，“宁夏著名商标”43个。

　　现代农业提质增效。预计2012年完成农业总产值89亿元，是2007年的2倍。“十大基地”示范带动效应增强。建成自治区级以上农业示范基地16个。孙家滩国家现代农业示范区成为全区节水农业样板，金银滩奶牛核心区成为国家农业产业化示范基地，红寺堡、青铜峡成为全国重要的酿酒葡萄种植基地。粮食总产90万吨，实现“九连增”。全区13个优势特色产业发展规模，我市保持着优质粮食、奶牛等8个第一和肉牛、红枣等5个第二的优势。塞外香大米、盐池甘草、同心圆枣、御马葡萄酒成为“农字号”知名品牌。累计认证无公害、绿色、有机农产品305个，认定国家地理标识18个。培育市级以上农业产业化龙头企业154家，农民专业合作组织506个。流转土地66万亩。

　　第三产业繁荣活跃。预计2012年实现社会消费品零售总额70亿元，是2007年的2.3倍，年均增长17.6%。实施市区商贸核心区改造工程，规划建设“十大市场”，打造了一批功能完善、特色鲜明的县级商圈、专业市场和特色街区。五年累计新改扩建各类专业市场30个。“万村千乡”、“家电下乡”等工程深入实施，累计建成标准化农家店963个。餐饮、商贸等传统服务业活力增强，连锁经营、电子商务、物流配送、金融保险等现代服务业蓬勃发展，苏宁电器、北京华联落户吴忠。中华黄河坛、黄河楼等文化旅游项目建成，红色旅游、航空文化游等特色旅游业加快发展。接待游客由2007年的117万人次增加到196万人次，旅游收入由3.6亿元增加到11亿元。吴忠市被评为中国优秀生态旅游城市。

　　——五年来，加快城乡发展，城乡面貌发生新巨变。彰显“黄河金岸·水韵吴忠”特色，城乡建设实现重大突破。五年共实施城乡建设项目1518项，完成投资733亿元，建设面积2806万平方米。全市城市建成区面积由2007年的50平方公里扩展到125平方公里，城市化率由39%提高到49%，是城乡建设项目最多、投入最大、速度最快、成效最显著的时期。

　　滨河新区绽放异彩。“两馆一中心”、黄河文化园、滨河体育运动公园等23个黄河金岸标志性工程建成。黄河文体会展中心、滨河新区商业城等顺利推进。金岸美地、恒大名都、黄河外滩等一批高品质居住区拔地而起。滨河大道、世纪大道、慈善大道、东兴大街、同心大街等建成通车，累计新建扩建市区城市道路160条360公里。实施黄河过境段综合整治、城市防洪排涝及清宁河、罗家河生态景观水道等重点工程，城市水面增加到8万亩，是2007年的11倍。东南部建设工程完成投资70亿元，城市框架进一步拉大。市区建成区面积由30平方公里扩展到80平方公里，城市化率由45%提高到56%。

　　城镇建设展现新姿。举全市之力掀起“两大工程”热潮。红寺堡城市道路与城南生态综合整治、盐池城市西区与城南新区开发、同心旧城拓改提升与新区建设等大县城建设全面推进，县城功能不断完善，面貌大为改观。金积、叶升、王团、惠安堡等特色小城镇旧貌换新颜。五年建成善谷小镇等塞上农民新居示范点20个，整治旧村庄369个，改造农村危房4万户。

　　基础设施日臻完善。太中银铁路、盐中、滚红、古青、211高速、城市黄河大桥、京藏高速吴忠出口改建等重大项目相继建成，全市公路通车里程达到5600公里，高等级公路突破1000公里，新修改造农村公路突破5000公里，大交通路网格局已经形成。刘家沟水库、花马池水库、清水河同心段治理等水利工程全面建成。城乡供热、供排水、供气、污水及垃圾处理、电网、通讯等基础设施建设取得重大进展。大力实施土地治理项目，耕地得到切实保护。高标准推进农田水利基本建设，农业基础不断夯实。利通区、青铜峡市、盐池县被评为全国农田水利基本建设先进县，我市农田水利建设创造了在全区“黄河杯”竞赛中五次夺冠、五次县县得奖、五获组织奖的历史最好成绩。

　　生态环境显著改观。三北防护林、退耕还林、封山禁牧、防沙治沙、天然林保护、湿地治理保护、山区小流域综合治理等工程深入实施，森林覆盖率、林木绿化率大幅提高。五年生态建设投入31亿元，营造林222万亩。全市有林面积达到520万亩，森林覆盖率达到13%，比2007年末增加3个百分点。城市绿化6.4万亩，城市绿地率、绿化覆盖率和人均公园绿地面积分别达到31%、36%和15平方米。生态建设群众发动程度、推进力度、投入资金、呈现效果实现历史性突破。吴忠市和青铜峡市、盐池县荣获国家园林城市（县城）称号。加大节能减排和水源地保护力度，淘汰关闭了一批污染企业和落后产能，实施农村环境连片整治和面源污染治理项目。“十一五”和各年度节能减排任务全面完成。

　　——五年来，深化改革开放，创新发展迸发新活力。“省管县”改革试点、红寺堡区设立和利通区理顺行政管理职能积极推进，乡镇机构改革全面完成。精简市直机关内设机构，实现人员编制动态化管理。推行监察机关派出监察分局管理模式和公安机关新型警务运行机制改革。对市直机关中层干部及工作人员实行交流轮岗。在全区率先建立保障和促进科学发展土地管理新机制。财税体制改革纵深推进，预算单位公务卡结算制度推广使用，化解乡镇债务2.2亿元。教育、文化、卫生等领域改革稳步推进。对外开放不断扩大，与阿根廷拉斯帕雷哈斯市、摩尔多瓦伯尔兹市缔结国际友好城市，同陕甘川蒙毗邻地区及中东部区域协作领域和层次不断拓展。坚持把“两大任务”作为推动经济社会发展的主抓手，2012年上争资金、招商引资实际到位资金分别达到102亿元、440亿元，增长50%和57.6%，分别是2007年的5倍和10倍。创造性地落实“黄河善谷”战略部署，率先提出慈善产业理念、创建慈善产业园区、建立残疾人职业培训基地、创建身障人士创业一条街、启动慈善城市创建工作，我市被评为全国七星级慈善城市、中国慈善年度推动者。成功举办奥运火炬传递、3届黄河金岸国际马拉松赛、第二十三届西交会、第二届全国城市公益慈善指数发布会等大型活动，对外知名度和影响力进一步提升。

　　——五年来，持续改善民生，人民群众共享新成果。五年用于民生投入达336亿元，占财政支出的82%。2012年承诺为民办理的10件实事全面完成，五年累计办理54件。养老、医疗、工伤、失业、生育保险制度全面建立，医疗、养老保险实现全覆盖。4.4万人社保历史遗留问题和10万被征地农民养老保险问题全面解决。征地拆迁补偿实现“零拖欠”。城乡低保实现应保尽保和提标扩面。持证残疾人免费体检、残疾人子女和残疾学生助学、残疾人白内障手术复明实现全覆盖，市残疾人康复中心、儿童福利院和流浪未成年人救助保护中心建成运行。五年累计发放全民创业贴息贷款10.7亿元，扶持建设各类创业园和孵化基地35个，培养小老板8100余名，新增就业岗位3.5万个。城镇“零就业”家庭实现动态清零，城镇登记失业率控制在4%以内。农村劳动力转移就业97万人次，建立了农民工工资支付管理机制。吴忠市被国务院授予全国创业先进城市。五年投入29亿元，新建、回购保障性住房2.7万套，发放租赁补贴7286万元，市区实现城市低收入住房困难家庭实物配租全覆盖。建成秦韵、开源广场、兴隆公园等一批公众休闲场所和高标准公交候车亭，对市区老旧小区住宅进行节能综合改造。“十二五”生态移民工程全面启动，投资14亿元，建设移民安置区14个，建成住房8517套，搬迁安置移民2.4万人。五年发放救灾款1.1亿元，救灾粮237万公斤。对157个贫困村实施“整村推进”扶贫开发，受益群众30万人。

　　——五年来，统筹协调发展，社会建设呈现新局面。城乡教育均衡发展。合理调整学校布局，全面实施校安工程，建成市区教育园区、同心县教育园区和一批中小学校，新建、改扩建校舍116万平方米，办学条件明显改善。深入开展“名师、名校、名校长”创建活动，顺利实现教育强县、“普高”目标。学前教育、职业教育、民族教育、特殊教育稳步发展。科技推广应用步伐加快。示范推广新技术110项、新品种106个，获得自治区级以上科技成果30项，科技进步奖26项。文化体育事业繁荣活跃。农家书屋覆盖所有行政村，农村卫星直播实现“户户通”。市图书馆、文化馆被评为国家一级馆。创作了《同心》、《罗山脚下》等一批优秀文艺作品，《曼苏尔》获“第二届全国戏剧文化奖”七项大奖，短篇小说集《换水》获第十届全国少数民族文学创作“骏马奖”。陈氏医技等8个项目列为自治区级以上非物质文化遗产传承保护基地。全民健身活动广泛开展，竞技体育取得新成绩。医疗卫生事业加快发展。公立医院改革试点全面推进，市医院通过三级乙等评审，与宁夏医科大学合作兴办吴忠中医院，实施了县、乡（镇）医院改扩建，村卫生室实现全覆盖。疾病预防、妇幼保健工作不断加强。青铜峡市、盐池县被授予国家卫生城市（县城）称号。人口自然增长率连续5年控制在10‰以内，我市成为全国婚育新风进万家活动示范市、全国人口和计划生育综合改革示范市。精神文明建设成果丰硕。自治区文明城市创建和民风建设活动扎实开展，总结提炼了“爱国、包容、和谐、奋进”的吴忠精神，涌现出海小平、李潇、纳振东等一批时代先锋。社会管理创新加强。全面贯彻党的民族宗教政策，深入开展“同心同行、同建同享”主题教育活动，启动全国民族团结进步模范市创建工作，利通区率先进入全国民族团结进步创建活动示范县行列。依法加强宗教事务管理，吴南大寺等4所宗教场所被命名为全国“和谐寺观教堂”。“平安吴忠”建设扎实推进，矛盾纠纷排查化解机制进一步健全，社会治安综合治理和防控能力大为提升，安全生产、食品药品安全、社会治安形势良好。妇女儿童、老龄、外事侨务、气象、档案、史志、统计、地震、住房公积金等工作有了新发展，国防教育、民兵预备役工作取得新成绩，实现全国双拥模范城“两连冠”。

　　——五年来，加快职能转变，自身建设得到新加强。依法治市方略得到有效落实，完成“五五”普法，启动“六五”普法。自觉接受人大法律监督和政协民主监督，认真办理人大代表议案、建议和政协委员提案，加强与民主党派、工商联、工青妇等人民团体联系。修订完善《市政府工作规则》和《市政府常务会议议事规则》，形成了征询公众意见、专家学者论证、依法进行审核、集体讨论决定的决策机制。营造风清气正发展环境活动成效明显。市政务服务中心迁建运行，规范了服务行为，方便了群众办事。深入开展纠风治乱专项治理和政风行风评议活动，“百姓直评干部”活动经验在全国推广。政府效能建设不断加强，连续两年获得全区效能考核一等奖。集中开展工程建设领域、“三公经费”、“小金库”等专项治理。政府采购五年节约财政资金2.1亿元。加强政府廉政建设，强化行政监察和审计监督，干部勤政廉政意识明显增强。

　　在肯定成绩的同时，必须清醒看到我们面临的困难和问题。主要是：经济实力弱，发展活力不足，“吃饭财政”的局面没有根本改变，保障发展的能力不强；产业层次低、规模小，可持续发展的基础还不牢固；城乡山川差距没有缩小，基础设施仍然薄弱，生态环境依然脆弱，县域经济发展不平衡；城乡居民收入水平较低，扶贫攻坚任务还十分艰巨；公共服务能力还不能满足人民群众需要，保障和改善民生还需要下更大气力；影响社会和谐稳定的因素较多，加强和创新社会管理工作任务繁重；政府自身建设还需不断加强，一些部门和工作人员作风仍需不断改进，发展环境有待进一步优化等。对此，我们将通过深化改革和加快发展，切实加以解决。

　　各位代表！回顾本届政府的工作历程，我们面对困难和问题，勇于实践，迎难而上，取得了令人鼓舞的成绩。这是自治区党委、政府和市委坚强领导的结果，是市人大、政协监督支持的结果，凝聚着全市人民的智慧和汗水，离不开社会各界的关心与支持。在此，我代表市人民政府，向全市各族人民，向人大代表、政协委员、民主党派、工商联、人民团体、离退休老同志和社会各界人士，向驻吴人民解放军、武警和消防官兵、政法干警，向中央及区属驻吴单位，表示衷心的感谢并致以崇高的敬意！

　　五年的工作实践，我们深刻地认识到：吴忠作为西部欠发达地区、民族地区、革命老区，要实现和保持经济社会又好又快发展，最核心的问题是一心一意谋发展、聚精会神搞建设，任何时候、任何情况下都不能有丝毫懈怠！最紧迫的任务是决不能放松项目建设，决不能放松产业发展，始终把“两大任务”作为加快发展的第一引擎！最根本的目的是不断改善民生，把人民群众的冷暖安危、福祉实惠、诉求祈盼系在心上、抓在手上、落到行动上！最有效的举措是解放思想、改革创新、敢于担当、攻坚克难，营造风清气正的发展环境！最关键的保证是党的坚强领导、人大政协的有力监督、人民群众的拥护支持、各级干部的真抓实干、社会各界的团结奋进！

　　各位代表，这些被实践证明行之有效的做法和经验，是科学发展观在吴忠的生动体现，是宝贵的精神财富，我们要倍加珍惜，一以贯之地坚持和发扬。

在全面建成小康社会进程中再创新辉煌

　　党的十八大提出了全面建成小康社会的宏伟目标和“五位一体”的总体布局，自治区十一次党代会作出了建设和谐富裕新宁夏、与全国同步进入全面小康社会的总体部署，市四次党代会明确了建设宁夏“五区”的奋斗目标。今后五年，是吴忠实现“五区”目标、全面建成小康社会的决定阶段、关键时期。在这样一个新的历史方位上开启新的征程，我们责任重大，使命光荣，信心百倍！

　　实现“五区”，建成小康，信心来自于对机遇的全面把握。总体看，世界经济复苏明显放缓，国内经济下行压力加大，但我国仍处于可以大有作为的重要战略机遇期，经济发展长期向好的趋势没有改变。全局看，十八大提出全面建成小康社会，优先推进西部大开发，加大对革命老区、民族地区、贫困地区的扶持力度。国家继续实施积极的财政政策和稳健的货币政策，更加突出“内需主导”，促进投资稳定增长。区内看，自治区进一步加大实施“两大战略”、“两区”建设、“黄河善谷”等工作力度。全面建成小康社会，全国的重点难点在西部，宁夏的重点难点在吴忠。重点会引起关注，难点能获得支持。这些战略部署，为我们加快发展创造了叠加机遇。

　　实现“五区”，建成小康，信心来自于对优势的充分发挥。吴忠地处引黄灌区的精华地带，国土面积占宁夏的三分之一，土地资源、光能风能资源、农产品资源和劳动力资源等优势明显，是呼包银经济带的辐射区，宁蒙陕甘毗邻地区的重要节点，沿黄经济带的核心区。近年来，全市综合实力大幅提升，县域经济实力不断壮大，经济内生动力显著增强，基础设施及生态建设取得重大进展，民生保障水平不断提高。吴忠的知名度、美誉度大幅提升。这些优势特色，为我们加快发展奠定了坚实基础。

　　实现“五区”，建成小康，信心来自于对挑战的积极应对。吴忠是西部欠发达地区，经济总量不大，财政实力不强，居民收入不高，消费能力不足，尤其是农民缺乏增收的产业和稳定的收入。全面小康社会实现程度低于全国、全区平均水平，目前位于全区五市第三。按照国家新的扶贫标准，还有24万贫困人口。我们总体上仍处在爬坡赶超的紧要关头，加快发展、实现跨越的压力和挑战依然紧迫。压力催生动力，挑战蕴含机遇。要清醒自信地认识到这些压力、挑战为我们加快发展带来的巨大精神力量。只要我们充分发挥出吴忠干部群众业已形成的奋发有为、敢为人先、激情干事、勇往直前的胆识和魄力，集中智慧，激发活力，凝聚力量，就没有战胜不了的困难，就一定能够赢得主动、赢得先机、赢得未来。

　　各位代表，今后五年政府工作的总体要求是：坚持以邓小平理论、“三个代表”重要思想、科学发展观为指导，认真贯彻落实中央、自治区和市委的决策部署，坚持稳中求快的总基调，以科学发展、跨越发展为主题，以加快转变经济发展方式为主线，以产业和项目为重点，以“两大任务”为突破，更加注重拓展发展空间，更加注重提高发展质量，更加注重激发发展活力，更加注重共享发展成果，更加注重社会和谐稳定，推动经济社会又好又快发展，为打造宁夏“五区”、建设和谐富裕新吴忠，全面建成小康社会而奋斗！

　　主要目标是：力争实现“六个翻番”。即：到2017年，地区生产总值、公共财政预算收入、城镇居民人均可支配收入、农民人均纯收入、工业增加值、全社会固定资产投资在2012年基础上实现翻番。率先在全区建成宁夏沿黄经济崛起区、城乡统筹发展样板区、生态文明示范区、民族团结进步模范区、黄河善谷和内陆开放型经济试验先行区。

　　——在做大做强产业经济上实现重大突破。产业经济是实现“五区”、建成小康的关键所在。今后五年要坚持走新型工业化、信息化、城镇化、农业现代化道路，推动信息化和工业化深度融合、工业化和城镇化良性互动、城镇化和农业现代化相互协调，促进工业化、信息化、城镇化、农业现代化同步发展。大力实施“三产八带”发展战略，进一步完善产业规划、优化产业布局、找准产业定位、突出产业集群、做大产业规模，实现速度、质量、效益提升。一是加快建设三条工业经济带。以太阳山开发区为核心，盐池县高沙窝镇和同心县韦州镇为两翼，依托庆华、太阳镁业等骨干企业，重点发展煤化工、新能源、新材料等产业，着力建设东线盐太同工业经济带，打造西北重要的能源化工基地。以慈善大道为轴线，连接立德、弘德慈善产业园区，依托伊利、夏进乳业、宝迪食品、吴忠仪表、弘德包装等骨干企业，重点发展食品加工、装备制造、印刷包装等产业，着力建设中线立弘慈善工业经济带，打造中国重要的自动化控制装备和慈善兴业基地。以新材料基地为核心，依托青铝、大唐、青龙管业等骨干企业，重点发展铝镁深加工、能源电力、化工建材等产业，着力建设西线沿山工业经济带，打造引领战略型新兴产业高地。到2017年，力争培育年销售收入过100亿元的大型企业2家、过50亿元企业2家、过20亿元企业7家、过10亿元企业10家。能源化工、新材料、装备制造、食品加工四大产业总产值突破1000亿元。二是加快建设四条农业示范带。以孙家滩国家级现代农业科技示范区为核心，重点发展奶牛、设施农业、精品果蔬、优质牧草等产业，着力建设罗山大道现代农业示范带，打造西部最大的奶牛养殖基地。依托利通区、青铜峡现代农业优势，重点发展优质粮食、酿酒葡萄、适水产业，着力建设黄河金岸高端农业示范带，打造中国优质大米基地。依托“同心圆枣”品牌和枸杞基地，重点发展圆枣、枸杞、中药材等特色产业，着力建设同心东部旱作农业示范带，打造西北旱作节水高效农业示范基地。依托中国“滩羊之乡”、“甘草之乡”品牌，重点发展滩羊、优质牧草、中药材特色产业，着力建设盐池中部生态畜牧业示范带，打造西北畜牧养殖加工示范基地。到2017年，培育奶产业、粮油加工、牛羊肉等3个“百亿元”产业集群。农业总产值突破100亿元。三是加快建设文化旅游产业带。以黄河为主线，依托黄河楼、108塔、中华黄河坛等资源，建设吴忠黄河文体会展中心、黄河文化展示园、大禹文化园和黄河动物园、植物园、游乐园等一批地标性景点，着力建设沿河文化旅游产业带，辐射带动红色文化游、生态农家游等特色旅游业。到2017年，旅游收入突破25亿元，将“黄河大峡谷·中华黄河坛”打造成国家5A级景区。

　　——在统筹城乡建设上实现重大突破。城乡建设是实现“五区”、建成小康的关键环节。今后五年要大力实施自治区沿黄经济区发展战略，推进“1个主城区、3个大县城、20个中心镇、200个新型社区”建设。一是加快利青同城化进程。统筹抓好利通区、青铜峡城市道路、绿化、水系等基础设施建设，不断完善城市功能，全面提升城市品味，提高承载能力。推进商贸、金融、医疗、通讯、交通等居民生活必需的服务网点向黄河两岸集中。到2017年，建成区面积达到90平方公里以上，城市人口达到50万人以上。二是加快建设大县城。根据盐同红县情特点，抓好规划、突出特色、发挥优势，加快推进基础设施建设，进一步完善物流、产业等要素聚集，形成县域中心。红寺堡建成新型生态移民城，盐池县建成森林县城，同心县建成中部干旱带生态商贸县城。三是加快建设新农村。提升中心集镇聚集辐射功能，推进农村社区化进程。进一步完善大村庄规划和基础设施建设，逐步拆迁、整合零散庄点，推进公共服务向农村延伸。加快中心镇和农村新型社区建设，建成一批功能完备、带动力强的特色村镇和新型社区。

　　——在扩大内陆开放成效上实现重大突破。内陆开放是实现“五区”、建成小康的难得机遇。今后五年要着力完善规划、突出项目、错位发展。坚持不懈地抓好“两大任务”的落实，上争项目、招商引资年均增幅保持在20%以上，率先建设内陆开放开发核心区。一是深化重点领域改革。创新对外开放体制机制，创造条件，力促一批企业和产品进入银川保税区。加大公共预算投资领域改革，强化市场运作，建立多元化的投入机制。加快户籍制度改革，完善农村基本经营制度，壮大村集体经济。二是打造宁夏向西开放的重要城市。积极争取在蒙古国边界建立沿边口岸，参与宁蒙陕甘能源化工“金三角”和呼包银经济区合作，强化分工协作，优势互补，资源互济。三是打造具有较强吸引力的承接产业转移示范区。进一步营造承接产业转移的体制环境，强化闽宁对口扶贫产业转移衔接，积极推进与中东部发达地区、国内大企业集团联合办园，政府间“飞地”建园，不求所有，但求所在，实现共建共享，合作共赢。

　　——在切实改善民生上实现重大突破。改善民生是实现“五区”、建成小康的根本目的。今后五年要千方百计提高城乡居民收入，进一步缩小收入分配差距，实现基本公共服务均等化，社会保障全面覆盖，社会就业更加充分，贫困人口大幅减少。一是突出民生财政、民生预算。坚持“强市”与“富民”同步发展，大力实施民生计划，努力保持每年公共财政投入80%以上用于民生事业，使人均基本公共服务接近全国平均水平。二是实施收入倍增计划。推进全民创业、家庭创业、自主创业。一手抓财政增长，一手抓居民增收，提高劳动报酬，完善分配机制，多渠道增加居民财产性收入，增加低收入者收入。到2017年，城镇居民可支配收入、农民人均纯收入力争实现翻番。三是完善社会保障体系。以人人享有生活保障为目标，城乡居民养老、医疗保险参保率分别达到90%和98%以上。五年城镇新增就业人口8万人，转移农村劳动力80万人次。积极创造条件，解决他们在养老、医疗、就业创业方面的后顾之忧。四是办好人民满意的教育。大力发展民族教育、职业教育、学前教育，办好特殊教育，支持民办教育，推进义务教育均衡发展。优化教育布局，扩大优质资源，提高教育质量，满足人民群众多样化的教育需求。五是提高人民健康水平。健全市、县、乡、村（社区）四级医疗卫生服务网络，提高医疗救治和康复服务水平，解决群众看病难、看病贵问题。加强食品药品监管，确保饮食用药安全。提高出生人口素质，稳定低生育水平，促进人口长期均衡发展。六是推进生态移民和扶贫开发攻坚。全面实施自治区百万贫困人口扶贫攻坚战略，用好六盘山集中连片特殊困难地区扶贫开发攻坚机遇，完成“十二五”1.6万户7.2万移民搬迁安置任务。深入推进移民新村建设、产业培育、公共服务、技能培训、社会管理等工作，教育引导移民群众自力更生、勤劳致富，解决好出路、发展、增收问题。健全扶贫工作长效机制，广泛开展社会帮扶，加强闽宁对口协作，加快贫困村“整村推进”步伐，扩大村级互助资金覆盖面，不断提高贫困群众自我发展能力。

　　——在加强生态文明建设上实现重大突破。生态文明建设是实现“五区”、建成小康的基本任务。今后五年要奋力推进生态文明建设，将之融入经济、政治、文化、社会建设各个方面，加快推进生态城市、生态农村和生态工业建设，让吴忠天更蓝、地更绿、水更清、城更美。生态城市以黄河金岸为重点，突出扩大绿地水面、提质增景，打造集绿化美化、生态旅游为一体的沿黄城市带景观长廊。县城集镇加快构建环城环镇大绿化格局。生态农村抓好农田林网、村庄绿化、封山育林、防沙治沙、面源污染治理等生态工程，继续大力开展农田水利基本建设。高度重视和切实抓好水源地规划、开发、保护和利用。做好生态移民迁出地生态保护和生态恢复。健全城乡环境综合整治长效机制。生态工业严格执行“十大铁律”，加强节能减排，推广清洁生产，大力发展循环经济、低碳经济。到2017年，万元GDP能耗下降15%，化学需氧量、氨氮、二氧化硫、氮氧化物分别削减6%、8%、4%和5%。森林覆盖率达到17%，城市绿地率、绿化覆盖率、人均公园绿地面积分别达到35%、38%和17平方米。吴忠市进入国家卫生城市、国家环保模范城市行列，红寺堡区、同心县建成自治区园林城市。

　　——在拓展提升慈善产业上实现重大突破。发展慈善产业是实现“五区”、建成小康的创新举措。今后五年要奋力推进慈善产业发展，改善残疾人等弱势群体生产生活条件，打造招善引资的政策洼地、践行社会责任的道德高地和投资兴业的理想之地。加快慈善产业园区建设，完善基础设施，增强服务功能，将慈善园区建成招善的金字招牌、亲善的合作平台、安善的创业基地。依托“黄河善谷”残疾人职业教育培训基地和各类培训平台，加强对有劳动能力残疾人的技能培训，增强受助人群自我发展本领。积极推进慈善城市创建活动。力争每年引进50个以上重点项目落户慈善产业园区。到2017年，慈善产业产值达到200亿元以上。全市有劳动能力的残疾人就业率达到80%以上，无劳动能力残疾人供养率达到70%以上。

　　——在发展繁荣文化上实现重大突破。推进文化大发展大繁荣，是实现“五区”、建成小康的重要内容。今后五年要全力构建覆盖全社会的公共文化服务体系，形成与经济社会发展相协调的文化发展格局。一是积极培育社会主义核心价值观。大力倡导富强、民主、文明、和谐，倡导自由、平等、公正、法治，倡导爱国、敬业、诚信、友善。广泛开展群众性精神文明创建和民风建设活动，教育引导公民形成积极向上的精神追求和健康文明的生活方式。二是大力发展文化事业。加快县（市、区）文化场馆、乡村文化场所、文化镇村建设，全面构建覆盖城乡、惠及全民的公共文化服务体系。大力开展形式多样的群众性文化活动，扶持发展民间文艺团队。广泛开展全民健身活动。三是加快发展文化产业。深入挖掘中华优秀文化、红色经典文化、黄河农耕文化，培育地方特色文化产业。鼓励社会各界兴办文化实体，积极发展演艺娱乐、文化创意和文化会展等产业形态，培育一批有特色、有影响、有活力的中小文化企业。加强文化市场建设。四是加强宣传和舆论引导。增强驾驭和运用新兴媒体能力，加强网络监督，做好舆情信息管理，促进网络文化健康发展。推动广播、电视、新闻出版事业发展。到2017年，进入创建全国文明城市先进城市行列。

　　——在创新社会管理上实现重大突破。创新社会管理是实现“五区”、建成小康的重要保障。今后五年要全力加强社会管理体制机制、能力体系、人才队伍和信息化建设，不断提高社会管理科学化水平。改进政府提供公共服务方式。加强基层社会管理和服务体系建设，增强城乡社区服务功能，强化企事业单位、人民团体在社会管理和服务中的职责。完善和创新流动人口和特殊人群管理服务。完善信访制度和人民调解、行政调解、司法调解联动的工作体系，畅通和规范群众诉求表达、利益协调、权益保障渠道。健全劳动关系协调机制，构建和谐劳动关系。信访案件处理率和信访群众满意率均达到95%以上。深化“平安吴忠”建设，加强社会治安综合管理，群众对社会治安状况满意率达到90%以上。建立健全重大决策社会稳定风险评估和各项安全生产监管机制，遏制重特大安全事故发生。全面落实党的民族宗教政策，加强宗教事务依法管理，指导协调做好生态移民村宗教活动场所规划建设，及时妥善处理涉及宗教领域的各类问题。到2017年，建成全国民族团结进步模范市。

　　各位代表，实现“五区”目标、全面建成小康社会，是时代的要求、人民的期望。我们坚信，经过五年的努力，一个更有实力、更具活力、更富魅力、更加美丽的新吴忠必将展现在世人面前！

2013年主要工作

　　2013年，是全面贯彻落实党的十八大精神的第一年，是新一届政府履职的起步之年。按照市委四届四次全体（扩大）会议的总体部署，明年全市经济社会发展主要预期目标和约束性指标是：地区生产总值增长12%以上；公共财政预算收入增长17%以上；固定资产投资增长30%以上；社会消费品零售总额增长15%以上；城镇居民可支配收入、农民人均纯收入分别增长12%以上；人口自然增长率控制在9.5‰以内；城镇登记失业率控制在4%以内。完成自治区下达节能减排目标。明年政府工作的主基调是：围绕“两大任务”，狠抓项目建设，狠抓产业发展，狠抓民生改善，狠抓环境优化，狠抓效能提升。突出抓好以下八个方面的工作：

　　（一）强化工业主导地位，全力提升工业经济发展实力。坚定不移实施“兴工强市”战略，加速新型工业化，进一步做大增量、调优存量，促进经济结构转型升级，确保规模以上工业增加值增长15%以上。力争年内新建、续建投资2000万元以上重点工业项目140个，完成固定资产投资180亿元，建成重点项目50个。强力推进三条工业带项目建设。完成年度计划编制实施工作，优化项目布局。盐太同工业经济带重点加快庆华二期和通达两个110万吨煤焦化、太阳镁业10万吨硅铁、山泰风机塔筒及钢结构制造、金裕海C4深加工等34个项目建设，建成投产24个。立弘慈善工业经济带重点推进国电热电联产、共创200兆瓦硅基薄膜、弘德包装40万箱烟包印刷等41个项目建设，建成投产26个。沿山工业经济带重点实施大坝四期2×100万千瓦火电、恒源冶炼公司矿热炉、昆宁公司氢氧化钡等15个项目建设，建成投产8个。强力推进“五优一新”产业振兴。抓好万胜生物600吨D —核糖、庆华50万吨甲醇制芳烃等30个“五优一新”项目，推进夏进液态奶扩建等25个重点技改项目，实施青铝电解槽能量优化等一批重点技术创新和新产品试产项目。支持吴忠仪表、吴忠卷烟等本地优势企业加快发展，打造行业知名企业和地标性品牌。积极扶持小微企业发展，培育一批内生性强、成长性好的中小企业群体。强力提升园区发展水平。继续把工业园区作为支撑工业发展的主阵地，进一步提升承载、服务和招商能力，不断提高园区产业集中度和规模化水平。强化市级统筹，实施分类指导，鼓励和支持各县（市、区）打造特色园区、专业园区。市财政安排2000万元专项资金，用于扶持重点企业和园区建设。切实加大节能减排力度。抓好50户重点企业节能降耗和41户重点企业污染治理，加快电厂、水泥行业脱硝工程、盐同红生活污水处理厂、农村面源污染治理等项目建设，坚决关闭淘汰不符合国家产业政策的企业和落后产能。

　　（二）加快现代农业进程，全力拓展农民增收致富空间。大力推进“两大农业示范区”建设，促进现代农业上规模、提水平、增效益。加快四条农业示范带建设。争取将“四带”建设列入自治区现代农业发展总体规划。罗山大道现代农业示范带重点加快孙家滩、五里坡等规模化奶牛、肉牛牧场和精品果蔬基地建设，新增奶牛存栏2万头、肉牛饲养量4万头、果蔬5万亩、酿酒葡萄2万亩。黄河金岸高端农业示范带加快叶升地三现代农业示范园等一批种植示范基地建设，确保优质粮食稳定在100万亩，设施农业总面积达到17万亩，有机富硒水稻达到12万亩。同心东部旱作农业示范带新增枸杞2万亩、红枣1.5万亩、中药材3万亩，建设马铃薯标准化示范基地4.5万亩。盐池中部生态畜牧业示范带力争滩羊饲养量突破300万只、优质牧草10万亩、甘草20万亩。提升农业产业化发展水平。实施“龙头企业升级工程”，支持伊利、夏进、中航塞外香、天予枣业等龙头企业，走精品化、高端化、集约化发展之路。年内引进培育国家级龙头企业1家、自治区级8家，专业合作社10个。加快农业工厂化、标准化、品牌化建设，新认定无公害农产品20个，绿色食品50个，有机农产品10个。加大科技兴农力度。推进农业技术集成创新、科技成果转化应用，加快农村信息化建设。加快优势特色产业新品种、新技术、新装备的引进示范，建立优质粮食、设施农业、奶牛养殖、草畜产业等产学研示范基地10个，引进试验示范新品种30个以上，新技术20项。夯实农业发展基础。继续高标准开展农田水利基本建设。切实抓好苦水河防洪、小农水重点县、土地整理等工程，新增节水灌溉面积10万亩，发展高效节水面积5万亩，治理水土流失面积240平方公里。市财政安排2000万元专项资金用于扶持农业发展。

　　（三）推动服务业“二次创业”，全力促进第三产业蓬勃发展。把发展现代服务业作为调结构、转方式、促就业、增收入的重要举措，进一步增强发展活力。改造提升商贸物流业。加快CBD商务中心、“宏泰、金龙、罗湖、福林”四大商业广场等项目建设，推进以民族特色餐饮一条街为主的沿河旅游休闲商贸带建设，改造提升一批老旧商业场所。加快“十大市场”和县级专业市场建设与启动运营，形成立足吴忠、辐射周边的区域性物流集散地。抓好农超对接试点城市工作，深入实施“万村千乡市场”等流通惠民工程。大力发展社区服务、金融保险、商务会展等新型服务业。全面提升餐饮业水平。充分发挥传统餐饮优势，加快调整特色菜品结构，丰富地方菜系。加强规范管理，提升服务质量。大力培育特色餐饮品牌和优势企业，鼓励餐饮名店走出去，发展连锁经营，培育一批在全区、全国叫得响的餐饮名店和名小吃连锁店。推动旅游业大发展。加快完善中华黄河坛、黄河楼等景点设施。引进大企业，整合包装文化旅游产业带资源，推动旅游产业市场化。年内将“黄河大峡谷·中华黄河坛”创建为国家4A级景区。大力开发旅游产品，逐步推进旅游景点免费开放。举办感恩母亲河文化旅游节等大型活动。全年接待游客220万人次，实现旅游收入14亿元。市财政安排1000万元专项资金，用于扶持第三产业发展。

　　（四）加大城乡建设力度，全力推动城乡一体化发展。以实施沿黄经济区战略为契机，高标准、高水平建设和管理城市。全年实施城乡建设项目220项，其中市区重点建设项目46项，概算总投资203亿元，年内完成投资135亿元。全市城市化率达到51%，市区达到58%，分别提高2个百分点。推进滨河新区建设。加快黄河楼内装饰工程、黄河文体会展中心、兰花花五星级酒店等续建项目建设，完成投资13亿元。开工建设乃光湖湿地公园、城北休闲公园，完成投资3900万元。促进房地产业健康发展。新建续建恒大名都、黄河外滩、罗湖锦都等33个房地产项目，完成投资15亿元。加快完善滨河新区基础设施建设。提升大县城建设水平。红寺堡区启动城区饮水工程和集污管网体系建设，实施小康街等30公里市政道路改建工程。盐池县加快新区开发建设，实施市民文化广场、新区公园等项目，完成“五纵四横”主干道绿化和景观工程。同心县开工建设图书馆、博物馆、休闲运动广场等项目，完善新区道路、供排水等基础设施。加快新农村建设。加大“迁村并点”力度，全力推进沿黄城市特色村、小康示范村建设。加快金积、预旺等特色城镇建设，建成“塞上农民新居”示范点8个，整治旧村庄80个，改造农村危房1万户。加强生态环境建设。加快沿黄城市带绿色景观长廊建设,做好历史人物文化园等9处、利华街北段等17条道路绿化和提质增景，完成绿地建设面积4650亩。抓好盐同红中部干旱带防风固沙长廊建设，新增防沙治沙及荒山造林7万亩、封山育林13万亩。实施绿色通道、村庄绿化、农田林网等绿化项目，完成生态防护林2.5万亩。全市完成营造林30万亩。

　　（五）抢抓“两区”建设机遇，全力提升内陆开放开发水平。充分发挥人文、地缘、产业和民族团结优势，扎实推进“两区”建设。高标准编制实施规划。根据试验区总体规划，找准定位，主动与国家有关部委和自治区相关厅局对接。制定更加优惠的政策，先行先试，力求在食品加工、农产品精深加工等方面有所突破。全方位开展外引内联。丰富和提升回商大会内涵和举办方式，进一步扩大影响力和吸引力。鼓励、支持有条件的企业、高校在吴忠建立产品研发、设计、质检。加强与国际友好城市往来，推进务实合作。利用物联网等信息技术和“智慧吴忠”平台，提高外引内联水平。大力度推进项目建设。始终把“两大任务”作为加快发展的重中之重 ，紧盯内陆开放政策投向，积极承接中东部产业转移，确保落地一批大项目、好项目。集中开展项目建设大会战，重点实施经济、社会、生态、民生、文化等领域6大类727个项目，完成年度投资696亿元。加强对招商引资项目协调服务，不折不扣落实好招商承诺，进一步研究扶持政策，给予企业家更多的荣誉，发挥“政策洼地”的积极效应。全年落实上争资金116亿元，招商引资到位资金500亿元。市财政安排1200万元专项资金，用于“两大 任务”以奖代补。

　　（六）巩固慈善产业成果，全力拓宽创新发展路径。进一步优化政策、整合资源、完善体系，加快慈善产业发展。推进“三园”建设。重点推进立德园慈善大道与银平公路连接线道路、供水管线，弘德园变电站、园区10平方公里拓展区，同德园水电路、产业孵化园等基础设施建设，进一步增强园区项目集聚承载能力。提升招善成效。力促康龙酒业葡萄酒等一批签约项目尽快开工建设。实施更加优惠的政策，加大招善引资力度，力争年内引进50个重点项目落户慈善产业园区。策划组织举办慈善产业与慈善项目推介会等各类活动，吸引国内外慈善家、企业家及公益慈善组织到吴忠置业兴善。创建慈善城市。加快构建以“慈善超市”、“社区居民互助促进会”、“社区慈善义工协会”三位一体的慈善组织网络，加强慈善专业队伍、志愿者服务队伍建设。对全市4861名智力、精神、肢体一级持证残疾人，按照每人年均700元标准实施居家托养补助，让残疾人享受更多关爱。深入开展慈善“十进”、“善行”吴忠系列活动，加强慈善文化交流合作，努力打造“慈善吴忠”新名片。

　　（七）增强公共服务能力，全力提升民生保障水平。把百姓关心、群众期盼的民生问题放在政府工作的突出位置，明年市财政用于民生投入的资金不低于11亿元。年内全面完成15件民生实事。完善社会保障体系。加快推进统筹城乡居民养老、医疗保险工作，实现应保人群全覆盖。从明年起，市财政每年安排865万元，为全市60岁以上参加统筹城乡居民养老保险群众，每人每月增加10元基础养老金。进一步加大创业就业扶持力度，为市全民创业小额贷款担保中心注入担保基金400万元。新建保障性住房48万平方米9682套，实施棚户区改造1398户。继续抓好老旧小区节能综合改造。加快发展公交事业，在市区新增城市公交线路5条，公交车80辆。推进生态移民和扶贫攻坚。从明年起，市财政每年安排3000万元，用于生态移民搬迁和扶贫攻坚。年内新建移民住房3718套，搬迁安置移民4888户，为每户配置炉具一套。整合培训资源，开展移民技能教育培训。加强移民社会管理，做好移民村低保、儿童入学、社会治安、宗教事务、计划生育等社会管理工作，有条件的村设立警务室。加快社会事业发展。把立德树人作为教育的根本任务，全面实施素质教育，深化教育领域综合改革，着力提高教育教学质量。利通区、青铜峡市通过自治区义务教育均衡发展评估验收。市区新建城市幼儿园、小学、初级中学各1所。继续完善学生交通安全保障专线，对全市3900名家庭经济困难学生给予乘车补助。合理配置医疗资源，加强对外医疗合作，改进群众就医服务。完善重大疾病防控体系，提高突发公共卫生事件应急处置能力。年内力争完成市人民医院迁建主体工程。巩固婚育新风进万家活动示范市创建成果，稳定低生育水平。从2013年开始，对自治区确定的“少生快富”推进示范点新增项目中的两女户，市政府一次性奖励3000元，对其他新增整村推进示范点项目户一次性奖励1000元。实施国家免费孕前优生健康检查项目。加快文化事业发展。广泛开展丰富多彩的群众文化活动，全年 “送戏下乡”演艺惠民200场次，建成10家社区文化图书室，新建居民书屋100个。以创建国家级公共文化服务区为契机，积极争取文化产业项目。加快文化基础设施建设，推进董府修缮工程等重点项目建设。民俗博物馆、文化艺术馆、伊斯兰文化中心年内投入使用。推进文化精品建设，加快歌舞剧《曼苏尔》市场化步伐。办好第四届宁夏黄河金岸国际马拉松赛和第三届全市运动会。做好全国第三次经济普查。深入开展双拥共建，推进军民融合发展，加强国防后备力量建设。加强妇女儿童、老龄、外事侨务、气象、档案、史志、地震、住房公积金等工作。

　　加强财政税收工作。加大财源建设力度，拓展增收途径，积极争取中央和自治区转移支付，保障民生等重点支出。强化预算，严控支出范围和开支标准。加强税收征管，强化非税收入征缴，确保应收尽收。

　　（八）加强和创新社会管理，全力促进社会稳定和谐。强化党委领导、政府负责、社会协同、公众参与、法制保障的社会管理工作体系，切实提高社会管理科学化、法治化水平。加强民族宗教工作。开展“民族宗教工作科学化建设年”活动，深化民族团结进步创建工作，依法加强宗教事务管理，推进“和谐寺观教堂”创建活动。及时化解民族宗教领域矛盾纠纷，巩固民族团结、宗教和顺、社会稳定的良好局面。加强社会综合管理。开展重大决策社会评估工作，深化矛盾纠纷“大排查”、干部“大走访”等活动。加强信访初访办理，化解信访积案，畅通群众诉求表达渠道，健全纠纷协调、矛盾调处机制。加强社区阵地建设，将社区办公用房和居民公益性服务设施纳入城市建设总体规划和项目规划。严格落实安全生产责任，加强食品药品、道路交通、煤矿、危险化学品等行业监管，坚决防范重特大事故发生。加强社会舆情信息管理，强化对社会热点问题的正面引导。深入推进“平安吴忠”建设，加强社会治安管理，严厉打击各类违法犯罪，切实维护社会稳定。加强精神文明建设。大力倡导社会主义核心价值观，全面开展以爱国主义、诚实守信、职业道德、个人品德为重点的公民思想道德教育。营造“劳动光荣、创造伟大”的社会氛围，培育知荣辱、讲正气、作奉献、促和谐的良好风尚。大力弘扬和践行吴忠精神，深入开展“好人家、好商家”及“爱国敬业、诚信友善”主题实践等群众性创建活动，塑造政务诚信、商务诚信、社会诚信。

切实履行好人民赋予的神圣职责

　　新一届政府承载着人民的重托，肩负着富民强市的重任。我们必须以更加负责的态度、更加务实的作风、更加饱满的精神，努力建设对人民负责、受人民监督、让人民满意的政府。

　　践行执政为民的宗旨。牢记全心全意为人民服务的宗旨，把人民群众的呼声放在首位，坚持主动服务、优质服务，坚决治庸、治散、治懒、治奢，建设服务型政府。提升政务服务水平，完善“一门受理、并联审批、限时办结”工作模式，简化办事程序，提高服务质量，方便群众办事。进一步营造风清气正的发展环境，打造办事快捷、服务优质的政务环境。以新要求，展示新面貌！

　　提高依法行政的水平。自觉接受人大法律监督和政协民主监督，切实办好人大代表议案、建议和政协委员提案，把人大和政协的监督作为提升政府工作水平的有力保证。密切同各民主党派、工商联、无党派人士和工青妇等人民团体的联系，重视新闻舆论监督和社会公众监督。健全决策机制和程序，完善决策问责和纠错制度，推进政府科学决策、民主决策、依法决策。加强行政执法监督，切实做到公正文明执法。以新作为，拓展新气象！

　　增强推动发展的本领。切实抓好政府系统十八大精神学习，用十八大精神统一思想、指导工作，不断提高科学发展、驾驭全局和调处各种复杂矛盾的能力。创新思维，突破定势，用科学先进的理念、先行先试的胆识、行之有效的办法推动工作。对发展中的难点、焦点、瓶颈问题，主动应对，全力破解。以新举措，促进新发展！

　　弘扬求真务实的作风。坚决执行中央关于改进作风的“八项规定”，全面提升政府系统抓落实的能力。完善政府分工负责、协同作战、团队攻坚的工作体系和责任机制，确保政令畅通。坚持实事求是、真抓实干，摸实情、出实招、办实事、求实效。坚持雷厉风行、提高效率，开短会、说短话、戒空话、少发文。以新作风，树立新形象！

　　营造廉政勤政的正气。严格执行廉政建设各项规定，全面履行“一岗双责”责任制，健全政府系统惩防体系建设，进一步完善土地出让、政府采购、工程招投标等制度，加大重点领域、重大工程、重要环节审计监督和行政监察力度，从源头上预防腐败发生。严格按照政府工作规则办事，严格规范权力运行，增强行政工作透明度。严控行政开支，厉行勤俭节约。加大纠风和专项治理力度，坚决纠正行业不正之风。以新风气，开辟新局面！

　　各位代表！人民的事业光荣伟大，吴忠的未来前程似锦！让我们高举中国特色社会主义伟大旗帜，在自治区党委、政府和市委的坚强领导下，坚定信心，振奋精神，开拓进取，扎实工作，为打造宁夏“五区”、 建设和谐富裕新吴忠，全面建成小康社会而努力奋斗！
